# Hybridization with mountain hares increases the functional allelic repertoire in brown hares

**DOI:** 10.1038/s41598-021-95357-0

**Published:** 2021-08-04

**Authors:** Jaakko L. O. Pohjoismäki, Craig Michell, Riikka Levänen, Steve Smith

**Affiliations:** 1 Department of Environmental and Biological Sciences, University of Eastern Finland, Joensuu, Finland; 2 Department of Integrative Biology and Evolution, Konrad-Lorenz-Institute of Ethology, University of Veterinary Medicine, Vienna, Austria

**Keywords:** Evolutionary genetics, Population genetics, Ecological genetics, Evolutionary ecology, Invasive species

## Abstract

Brown hares (*Lepus europaeus* Pallas) are able to hybridize with mountain hares (*L. timidus* Linnaeus) and produce fertile offspring, which results in cross-species gene flow. However, not much is known about the functional significance of this genetic introgression. Using targeted sequencing of candidate loci combined with mtDNA genotyping, we found the ancestral genetic diversity in the Finnish brown hare to be small, likely due to founder effect and range expansion, while gene flow from mountain hares constitutes an important source of functional genetic variability. Some of this variability, such as the alleles of the mountain hare thermogenin (uncoupling protein 1, *UCP1*), might have adaptive advantage for brown hares, whereas immunity-related *MHC* alleles are reciprocally exchanged and maintained via balancing selection. Our study offers a rare example where an expanding species can increase its allelic variability through hybridization with a congeneric native species, offering a route to shortcut evolutionary adaptation to the local environmental conditions.

## Introduction

Understanding of the ecological and evolutionary processes underlying species’ dispersal and range limits is a fundamental theme in evolutionary biology^1^. The leading edge of the species range expansion is typically populated by few individuals, which are also living at the boundary of their specific habitat preferences. The genetic landscape of these boundary populations is influenced by founder effect as well as low population densities resulting in increased genetic drift and further loss of genetic diversity^2^. This low variability, could in principle, reduce the ability of the population to adapt to the local environment, slowing down the range expansion^1^.

**Figure 1:**
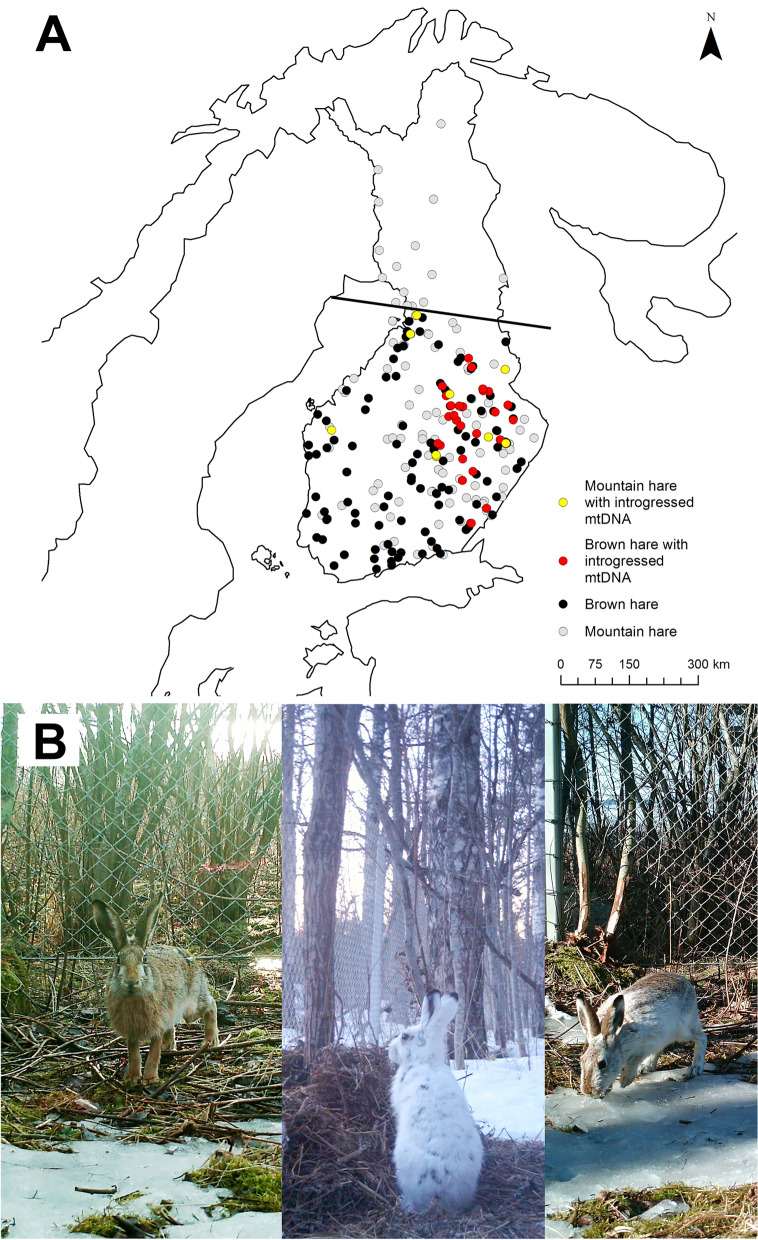
Hybridization among Finnish hares. (**A**) Distribution of samples used in the study. The level of mtDNA introgression roughly correlates with the expansion front of the brown hare^6^. The allopatric mountain hare samples north from the line were assumed as “purebred” for the purpose of the TSP analyses. Both species coexist south of the line, although mountain hare abundance decreases notably towards the south-west. (**B**) Excerpts from game camera images from the same location in Joensuu, Finland in April 2014. Typical brown hare in the left and mountain hare in the middle. Individual on the right was later captured and confirmed as a first-generation hybrid (sample 843), having brown hare mtDNA, but being heterozygous for the tested nuclear loci (Table S1). The individual was originally assigned as a brown hare based on morphology and mtDNA genotype.

Range expansions are often associated with introduced invasive species but there are also numerous examples of naturally occurring range shifts, especially under current global climate change^3^. This is also the case with brown hares (Lagomorpha: Leporidae, *Lepus europaeus* Pallas), native to Central Asia and mainland Europe, that are currently expanding their range northwards^4,5^ and have reached approximately the 65.3˚N in the Nordic countries^6^. The species arrived in Finland naturally from the south-east as early as the nineteenth century^7,8^, but has experienced an impressive 300 km range expansion to north in only the last 30 years, quite likely facilitated by climate change^9^. As brown hares frequently hybridize with the resident mountain hare (Lagomorpha: Leporidae, *Lepus timidus* Linnaeus) an arctic/subarctic species that has occurred in Fennoscandia for approximately 10,000 years^10^, we hypothesized that hybridization may have substantial evolutionary implications for the two species. In the present study, we compare levels of genetic diversity among the Finnish mountain hares and brown hares with Central European brown hares. Specimens from both Finnish hare species represent a hybrid population, with varying degrees of introgression, whose intensity roughly correlates with the invasion front of the brown hare’s northward expansion (Fig. 1). We are particularly interested in investigating whether the Finnish brown hares have obtained significant levels of adaptive allelic variants from mountain hares. In principle, the cross-species gene flow has the potential to allow the expanding species to co-opt beneficial, locally adapted alleles assisting brown hares to secure a foothold in the new environment. Adaptive advantage of the local alleles should manifest as overrepresentation of mountain hare alleles in brown hares that exceeds the levels expected from random genetic drift. In fact, previous studies have shown that hybridization with closely related species has provided both snowshoe hares (*Lepus americanus* Erxleben)^11–15^ and mountain hares^16–18^ with beneficial alleles related to winter pelage color. Due to the shorter snow-covered season, camouflage mismatch with white winter pelage on bare ground has been shown to carry fitness costs^19^, which cannot be compensated through adaptive behavioral plasticity^20^. Interestingly, it seems that natural selection can be too weak to adaptively shift the phenology of color molt in mountain hares^21^, underscoring the importance of obtaining adapted color morph alleles.

While the aforementioned studies have focused on how a local artic-alpine species might adapt to milder winter conditions due to climate changes, our interest was to detect potential adaptive gene flow to the expanding species. We chose four nuclear gene loci with known or expected evolutionary importance to serve as a proxy for adaptive introgression and contrasted these against the background introgression of mitochondrial DNA, assumably neutral nuclear gene as well as a panel of neutral SNPs for a subset of the samples. Our first candidate was thermogenin (uncoupling protein 1, *UCP1)*. UCP1 is a mitochondrial inner membrane channel protein, which channels protons, bypassing ATP-synthase and converting the energy stored in the mitochondrial membrane potential to heat instead of ATP^22^. UCP1-mediated heat production is especially important for small and adolescent mammals^23^. In fact, positive selection on certain *UCP1* variants has also been suggested to confer climatic adaptation in humans^24^. As an arctic/boreal species, mountain hare specific *UCP1* alleles are expected to be cold adapted in contrast to those of the brown hare, which originate from a much milder climatic region. Other evolutionarily significant genes are those of the major histocompatibility complex (MHC) class II *DQA* and *DQB* loci, which are critical for adaptive immunity. As MHC class II receptors are required for the pathogen recognition, a broader range of genetic variants in a population should offer protection against a broader range of pathogens and parasites^25^. As the brown hares at the species’ expansion front are likely to show little ancestral genetic variation due to the founder effect, the mountain hare/brown hare hybrids could benefit from the rare allele advantage at the MHC loci^26–28^, where the rare alleles have been obtained from the mountain hare. An additional benefit for analyzing the MHC loci is that they typically comprise many alleles in all populations, making them practical in estimating overall genetic diversity of a population^29–31^.

In addition, we analyzed Toll like receptor 2 (*TLR2*), a gene involved in innate immunity, whose allelic variants have been suggested to explain differences in pathogen resistance between various wild mammal populations^32–34^, as well as the succinate dehydrogenase subunit A locus (*SDHa*). *SDHa* encodes for a subunit of mitochondrial oxidative phosphorylation (OXPHOS) complex II (CII), which is the only OXPHOS complex encoded entirely by nuclear genes and therefore avoids any nuclear-mitochondrial incompatibility issues^35^. These in turn might cause mitochondrial DNA (mtDNA) driven selection on nuclear encoded mitochondrial genes. As a functionally conserved housekeeping gene, we expect the *SDHa* locus to be evolutionarily neutral and serve as a proxy for the introgression of nuclear genes in general.

The hybrid zone interaction between mountain hares and brown hares provides an ideal opportunity to compare the genetic diversity in a local vs. expanding animal population and to test if an expanding species can adopt locally adapted genetic variants from the resident species. In principle, this would allow a shortcut to evolutionary adaptation to the new environment^15^ as well as mitigate the effects of genetic drift at the leading edge of range expansion. We expected the expanding brown hares to be genetically less diverse due to founder effect and drift, as well as hypothesized that the mountain hare UCP1 alleles could represent local climatic adaptation that should confer a selection advantage in the Finnish hybrid zone brown hares. In contrast, MHC class II allelic diversity should be under balancing selection and thus broadly shared between the two species.

## Results

**Figure 2:**
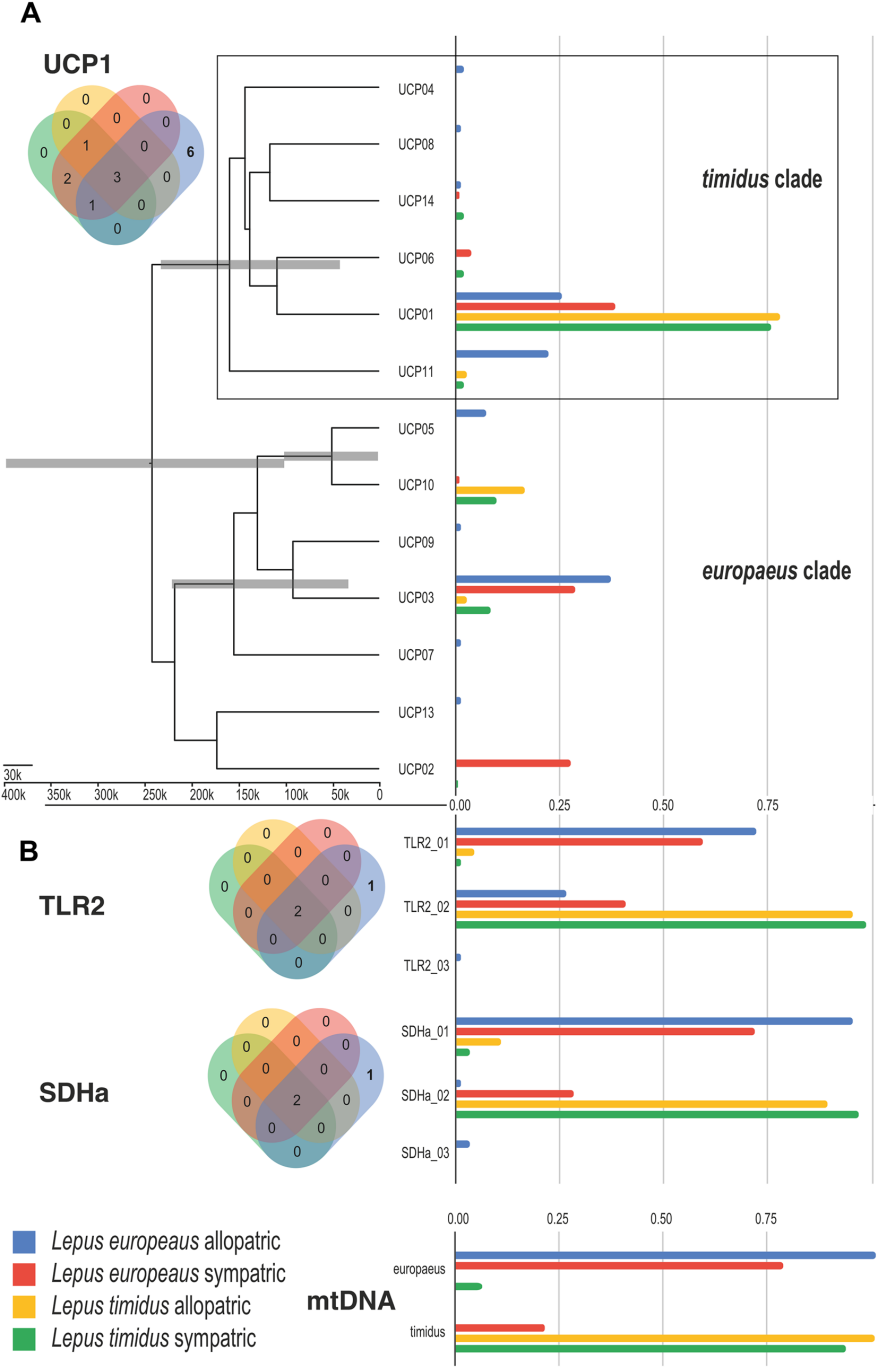
Ultrametric tree of *UCP1* and allele frequency bar chart of *TLR2* and *SHDa* allele frequencies and mtDNA haplotypes (*timidus* or *europaeus*) in hare populations. The bars over the tree nodes represent the 95% confidence interval of the node height. The colors of the allele frequency bar chart represent each of the populations tested. Venn diagrams depict the numbers of unique and shared alleles for each population (same coloring as for the allele frequency bar chart). Private allele numbers in bold.

**Figure 3:**
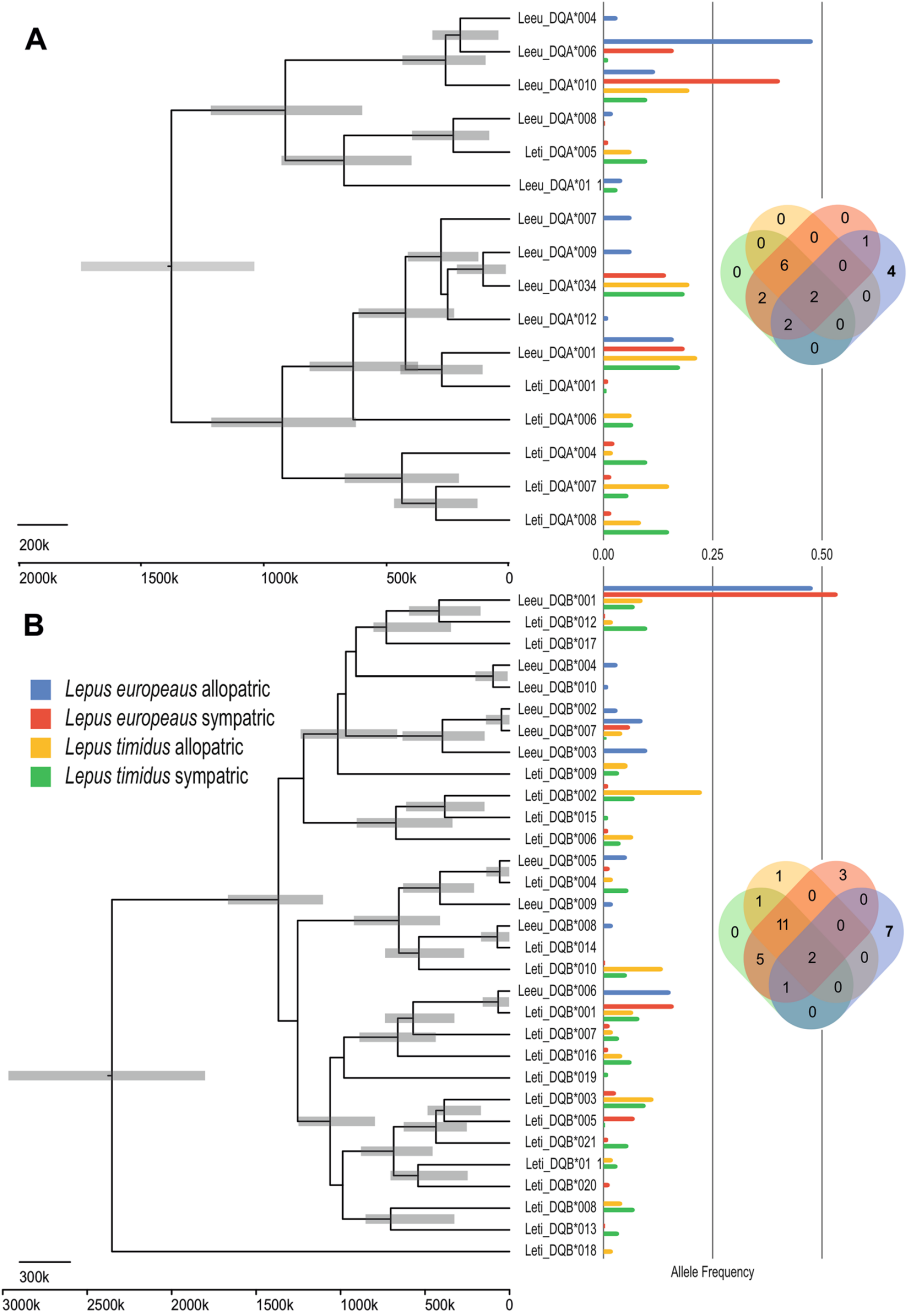
Ultrametric trees for the MHC allele relations and allele frequencies in Finnish mountain hares (*Lepus timidus*) and brown hares (*Lepus europaeus*) for assumed allopatric and sympatric populations. (**A**) *DQA* locus, (**B**) *DQB* locus. Tip labels of the tree represent the individual alleles in this study as identified by the following nomenclature, hare species_Locus*allele, for example, Leeu_DQA*001 is brown hare DQA locus 001. The scale of the ultrametric trees is in years from the present, based on a strict clock assumption of μ_DQA_ = 10% and μ_DQB_ = 11%. The bars over the tree nodes represent the 95% confidence interval of the node height. The colors of the allele frequency bar chart represent each of the populations tested. Due to the scale of the allele frequency not all bars are observable, for all allele frequencies refer to Table S3. Venn diagrams depict the numbers of unique and shared alleles for each population (same coloring as for the allele frequency bar chart). Private allele numbers in bold.

The overall genetic diversity (GD) as computed from the polymorphism data of all loci^36^ was 0.86 ± 0.67 for the Finnish mountain hares (n = 147) and 0.65 ± 0.45 for the Finnish hybrid zone brown hares (n = 173). Interestingly, GD was lowest (0.53 ± 0.28) for the allopatric Austrian brown hares (n = 48) used as a reference to detect brown hare specific variation. Much of the difference in genetic variation among Finnish hares can be attributed to the MHC class II genes, as *DQA* and *DQB* loci were dominated by one common allele each in brown hares (Leeu-DQA*006 = 0.49; Leeu-DQB*001 = 0.56), while mountain hares showed more even genotype representation and higher number of alleles compared to brown hares (Fig. 3, Table 1, Table S3). In addition, the levels of heterozygosity (*Hz*) were higher in mountain hares than brown hares for both MHC class II loci whereas the other nuclear loci showed higher *Hz* levels in brown hares (Table 1). Remarkable 83% and 70% of the *DQA* and *DQB* variation was explained by variation within individuals, whereas for *TLR2* or *SDHa* the variation was mainly between the species (Table 2). The introgression of mtDNA as well as *TLR2*, *SDHa* and neutral SNP alleles was notably different between the species, with 0.60–0.85 of the alleles being unique in brown hares, compared to 0.89–0.98 in mountain hares (Table 1). For both species, sympatric populations showed more allele sharing at all loci compared to allopatric populations (Fig. 2). Allele sequences have been submitted to GenBank and the accession numbers and project data are available online (see Data Accessibility).

**Table 1:** An overview of the of the genotyping results for 147 Finnish mountain hares (*Lepus timidus*) and 173 Finnish brown hares (*Lepus europaeus*) showing the number of genotype gene copies, alleles per locus, observed as well as expected heterozygosity frequency (*Hz*) and the statistical significance for the deviation from Hardy–Weinberg equilibrium (HWE).

***Lepus timidus***	***DQA***	***DQB***	***UCP1***	***TLR2***	***SDHa***	**mtDNA**	**GW SNPs**
Successful genotypes	138	140	121	138	141	147	17
Gene copies	276	280	242	276	282	147	6833 × 17
Alleles per locus	9	13	7	2	2	2	2
*Hz* obs	0.88	0.73	0.33	0	0.04	NA	0.15
*Hz* exp	0.85	0.78	0.41	0.04	0.09	NA	0.14
HWE *p*-value	NS	NS	< 0.001	NS	NS	NA	NS
Frequency of conspecific alleles	NA^#^	NA^#^	0.92	0.98	0.95	0.93	0.89^§^

***Lepus europaeus***	***DQA***	***DQB***	***UCP1***	***TLR2***	***SDHa***	**mtDNA**	**GW SNPs**
Successful genotypes	142	137	170	141	170	173	19
Gene copies	326	326	340	282	340	173	6833 × 19
Alleles per locus	10	14	7	2	2	2	2
*Hz* obs	0.68	0.61	0.50	0.08	0.24	NA	0.14 (mean)
*Hz* exp	0.75	0.67	0.70	0.48	0.42	NA	0.15 (mean)
H-W *p*-value	< 0.001	< 0.001	< 0.001	< 0.001	< 0.001	NA	NS (mean)
Frequency of conspecific alleles	NA^#^	NA^#^	0.56***	0.60**	0.72	0.77	0.85^§^

NS = not significant and NA = not applicable. For example, mitochondrial DNA (mtDNA) is haplotypic and therefore no *Hz* can be computed. Χ^2^ test against mtDNA introgression alone: ** *p* < 0.01, *** *p* < 0.001. ^#^No species-specific alleles could be assigned. ^§^Expressed as proportion of membership of pre-defined population (brown hare or mountain hare) in STRUCTURE analysis^86^ (See^45^). *GW* genome wide.

**Table 2:** Analysis of molecular variance (AMOVA) for each locus.

Locus	Source	df	Sum of squares	Expected mean squares	Est. Var	% of total molecular variance	Fst	Fis	Fit
DQA	Among Pops	3	19.30	6.43	0.038	8%	0.08 ***	0.09 ***	0.17 ***
	Among Indiv	364	161.63	0.44	0.036	8%			
	Within Indiv	368	137.00	0.37	0.372	83%			
	Total	735	317.92		0.446	100%			
DQB	Among Pops	3	23.22	7.74	0.094	19%	0.19 ***	0.14 ***	0.30 ***
	Among Indiv	364	167.42	0.46	0.056	11%			
	Within Indiv	368	128.00	0.35	0.348	70%			
	Total	735	318.63		0.498	100%			
UCP1	Among Pops	3	22.35	7.45	0.090	21%	0.21 ***	0.41 ***	0.53 ***
	Among Indiv	364	175.38	0.48	0.140	32%			
	Within Indiv	368	74.50	0.20	0.202	47%			
	Total	735	272.24		0.432	100%			
TLR2	Among Pops	3	59.54	19.85	0.251	55%	0.55 ***	0.67 ***	0.85 ***
	Among Indiv	364	124.78	0.34	0.137	30%			
	Within Indiv	368	25.00	0.07	0.068	15%			
	Total	735	209.32		0.457	100%			
SDHa	Among Pops	3	89.85	29.95	0.383	72%	0.72 ***	0.49 ***	0.86 ***
	Among Indiv	364	80.19	0.22	0.073	14%			
	Within Indiv	368	27.50	0.08	0.075	14%			
	Total	735	197.54		0.531	100%			

Populations used in this analysis have been partitioned as follows: brown hare allopatric, brown hare sympatric, mountain hare allopatric, mountain hare sympatric population. P-values are based on 999 random permutations. ***p < 0.001.

For the loci with more than three alleles across the entire dataset, we performed phylogenetic analyses to correlate the clustering of related alleles with their frequencies in the two species and four population groups (Figs. 2 and 3). We also estimated their divergence dates to compare the allele ages.

*UCP1, TLR2* and *SDHa* loci showed clear ancestral allele frequency differences (two clades, each dominated by one species) between mountain hares and brown hares (Fig. 2). Notably, the *UCP1* locus exhibited an allele appearing in high frequency in the Finnish hybrid zone brown hares, but not detected in either of the allopatric populations (UCP02). Evolutionarily however, this allele is well embedded in the brown hare clade but has presumably been lost or was unsampled in Austrian brown hares.

In contrast, the MHC loci showed high levels of variation as well as considerable allele frequency differences between species and population groups (Fig. 3A,B). Hybrid zone brown hare individuals retained most of the allelic variants found in both ancestral species and significantly higher levels of diversity than allopatric brown hares (compare the within individuals vs between populations variance in Table 2). As well as being a signature of introgression, the shared variation between mountain hares and brown hares is also typical for trans-species polymorphisms (TSP), which is the persistence of allelic lineages beyond speciation events and has often been associated with MHC loci^37^.

Hybrid individuals shared MHC alleles that occurred only in pure brown hares and alleles that only occurred in individuals assigned as “pure” mountain hares (Fig. 3A,B). Only two alleles each from *DQA* (Leeu-DQA*001 and Leeu-DQA*010) and from *DQB* (Leeu-DQB*001 and Leeu-DQB*007) were shared across allopatric and sympatric populations. There were however, nine additional *DQA* alleles and 13 *DQB* alleles that were shared only between hybrids and parental species. We take this as evidence that, despite detecting the expected signal of TSP, there is additional strong introgression of MHC alleles in both directions from the parental lineages. While many of the *DQA* alleles were previously known^38,39^, all of the *DQB* variants were novel discoveries in mountain hares and only three had been seen previously in brown hares (Table S3).

**Figure 4:**
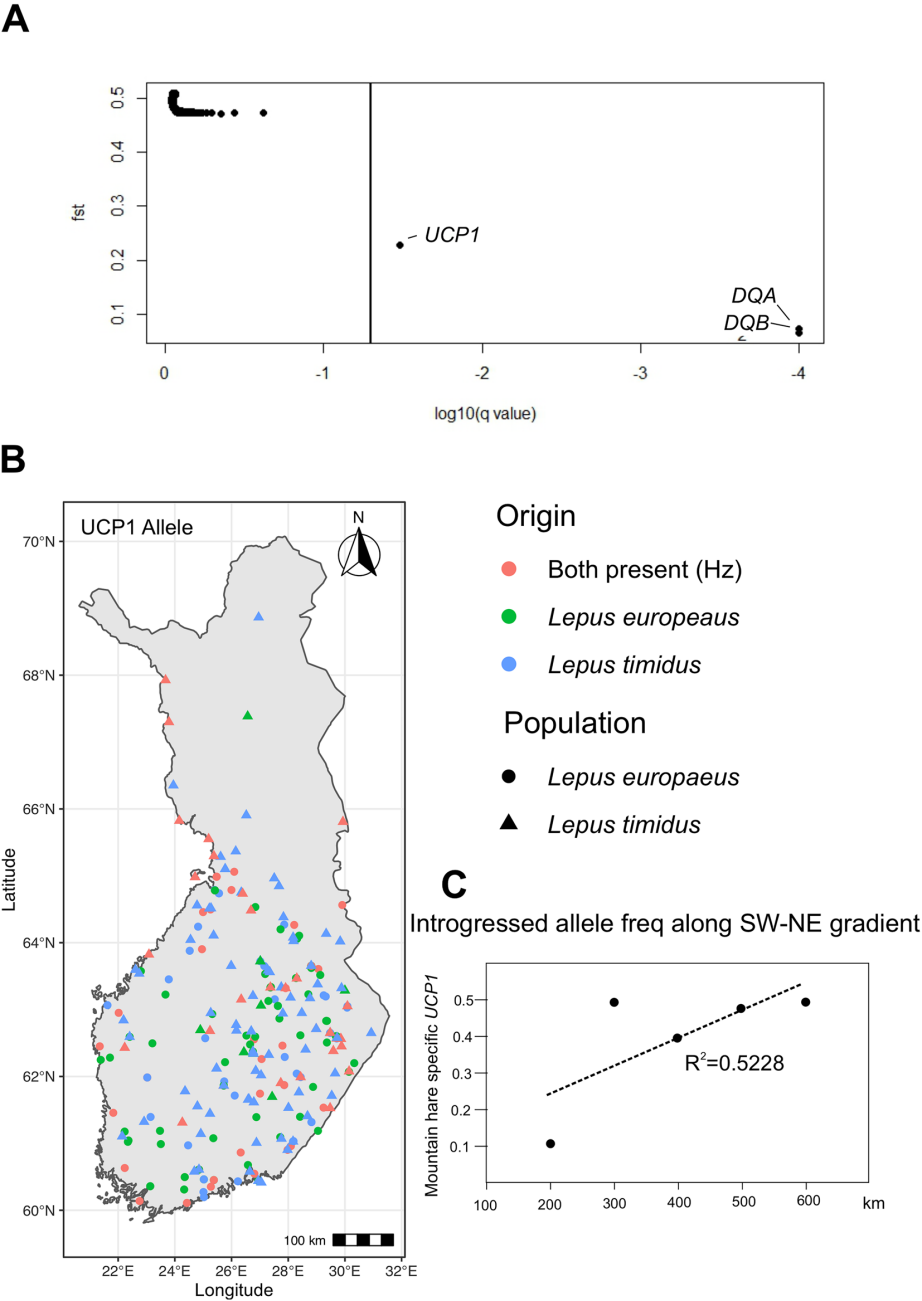
Potential selection advantage for *UCP1* and MHC allleles. (**A**) A graph of the results of the Bayesian outlier analysis for loci under selection showing evidence of a strong balancing selection acting on the two MHC genes (*DQA*: q = 0.0, *F*_*ST*_ = 0.074, α = − 2.76; *DQB*: q = 0.0, *F*_*ST*_ = 0.066, α = − 2.85) and moderate balancing selection for *UCP1* (q = 0.033, *F*_*ST*_ = 0.23, α = − 1.39). No selection was detected for *TLR2*, *SDHa* or the included SNPs (the remaining dots). (**B**) Mountain hare specific *UCP1* allele abundance in Finnish brown hares at each side of arbitrary SW-NE boundary, roughly following the median extent of 125 days of snow cover^6^. (**C**) Mountain hare specific *UCP1* allele frequency gradient along the south-west to north-east axis.

Coalescent simulations for locus-specific *F*-statistics^40^, showed evidence that *DQA*, *DQB* and *UCP1* were under selection in both species (Table S4). Similarly, the Bayesian outlier analysis (Fig. 4A) also revealed balancing selection acting on the two MHC genes (*DQA*: q = 0.0, *F*_*ST*_ = 0.074, α = − 2.76; *DQB*: q = 0.0, *F*_*ST*_ = 0.066, α = − 2.85) and moderate balancing selection evident for *UCP1* (q = 0.033, *F*_*ST*_ = 0.23, α = − 1.39). No selection was detected for *TLR2*, *SDHa*. Fitting with the role of UCP1 in cold tolerance, mountain hare specific alleles are more common in hybrids in the higher latitudes (Fig. 4B,C), although this tendency is not statistically significant.

## Discussion

The Finnish brown hare population has been growing in size and increasing its range northwards during the past few decades^6^. This development offers an interesting natural setting to study the population genetics and evolutionary adaptation of expanding species. The small pioneering populations at the edges of the distribution are influenced by founder effect and genetic drift, causing the edge populations to have lower genetic diversity than in the core populations^2^. As asymmetric gene flow from mountain hare to brown hare is well established^41–45^, we sought to see how this introgression contributes to the genetic makeup of the Finnish brown hares and whether the mountain hare alleles could have adaptive significance for the brown hares.

From our analysis, it was apparent that the genetic makeup of Finnish brown hares is heavily influenced by the introgression of alleles from mountain hare, at least for the analyzed loci (Table 1, Figs. 2 and 3). For example, Finnish brown hares had higher genetic diversity (GD) than their allopatric Austrian counterparts and the difference could be largely attributed to the representation of mountain hare alleles in Finnish brown hares. In contrast, Austrian brown hares had a number of private alleles in all analyzed loci, which were not present in the Finnish hares (Table 1, Figs. 2 and 3). If only the alleles that are shared between Austrian and Finnish brown hares (Fig. 2, Table S2) were taken into account, the GD of Finnish brown hares would be 0.40 ± 0.37. This is notably lower value than observed for the Finnish (0.65 ± 0.45) or Austrian (0.53 ± 0.28) brown hare populations. It should be pointed out that this is an estimate, as the Austrian population is not directly ancestral for the Finnish brown hares and because the origin of the MHC alleles cannot be traced based on their phylogeny. As another tell-tale sign of a founder effect, the most diverse loci *DQA* and *DQB* were dominated by one common allele each in brown hares (Leeu-DQA*006 = 0.49; Leeu-DQB*001 = 0.56), while mountain hares showed more even genotypic representation and higher number of alleles (Fig. 3, Table 1, Table S3), resulting also in higher levels of heterozygosity in mountain hares than brown hares for both MHC class II loci. In contrast, the other nuclear loci showed higher levels of *Hz* in Finnish brown hares, which can be explained by an asymmetric flow of alleles at these loci from mountain hares to brown hares, as alleles dominant in mountain hare were commonly present in brown hares but not vice versa (Table 1, Figs. 2 and 3, Table S3).

Taken together, these findings show that brown hares commonly obtain functional genetic variants from mountain hares and demonstrate that hybridization can increase genetic diversity in an expanding population.

Our prime candidates to detect potentially meaningful adaptive introgression in hares included the *UCP1* gene, which could have a potential role in adaptation for a colder climate^46,47^, MHC class II loci *DQA* and *DQB*, which confer general pathogen resistance and are known to be under strong balancing selection favoring genetic diversity in various chordates (e.g.^39,48–50^) and *TLR2*, required for innate immunity^51^. *SDHa* was expected to perform as a neutral marker, showing reduced evidence of adaptive introgression and mitochondrial DNA was included to track the maternal lineage hybridization of the individuals.

The conducted outlier analyses detected moderate balancing selection acting on *UCP1* and strong balancing selection at both MHC loci (Fig. 4A), while the maintenance of introgressed alleles at *TLR2* and *SDHa* likely reflects neutral processes similar to those at the genome-wide SNP loci (Levanen et al. 2018). This is also evident in the AMOVA analysis, which showed that the majority of the variation in neutral alleles was between the species, while the loci potentially under selection showed significant within-individual variation (Table 2). However, the balancing selection suggested by the Bayesian outlier analysis for *UCP1* likely points to some underlying dynamics of the hybridization and selection process. Alleles under purifying selection are identified from their tendency for fixation, while in the case of the Finnish brown hares, there is a constant input of ancestral alleles through hybridization, which are encountered by differential selection pressures. Revealingly, the mountain hare specific alleles were overrepresented in Finnish brown hares for *UCP1* and *MHC* loci compared to the neutral loci (Fig. 2, Table 1, Table S3). This results in rather even distribution of mountain hare alleles in the Finnish brown hares (Fig. 4B), which mimics the effects of balancing selection. In fact, it is well known that demographic factors of a population can specifically increase the false discovery of balancing selection in Bayesian outlier analysis^52^.

Our interpretation is that although some brown hare alleles were detected also in mountain hares, the dominance of mountain hare alleles in both species suggests that selection is favoring locally adapted alleles at the expense of outbred alleles per se. This is evident also from the fact that the Austrian allopatric brown hares have a surprising allelic diversity at the *UCP1* locus, including four alleles that are shared with the Finnish hares and six private alleles, while the *UCP01* allele is almost the dominant allele in both species in Finland (Fig. 2).

Adaptive thermogenesis in brown fat is dependent on the UCP1 and is essential for all neonate mammals^53^. At birth, the newly born cub needs to rapidly adapt from the maternal body temperature to the environmental temperature and this process is developmentally controlled. We chose to investigate the variation in *UCP1* specifically as both mountain hares and brown hares do not have nests or lairs but leave their young unattended after birth and typically feed the leverets only once per day. This means that especially the spring generation, often born on snow, is exposed to cold and variable weather conditions of a boreal climate and is independent of the mother for shelter or thermoregulation. Under these circumstances, any cold adapted alleles for NST are likely to make a difference for early season breeding for brown hares. Therefore, we find it credible that positive selection explains the excess of mountain hare *UCP1* alleles in the Finnish brown hare population. As most of Finland is at the northern edge of brown hare distribution, a steep allelic gradient of the mountain hare *UCP1* in the brown hare population is not necessarily expected (Fig. 4B,C). However, there can be also trade-offs with the cold-adapted alleles, such as metabolic inefficiency^54^, which could balance out the benefits when food is scarce or poor quality. We acknowledge that the evidence for the adaptive value of mountain hare *UCP1* alleles for brown hare is only circumstantial, and we can currently only hypothesize about the physiological differences between the different alleles, but these might be possible to investigate in future studies.

While balancing selection favoring allelic diversity is well established for MHC loci, the directionality of the gene flow between the two species is difficult to dissect. As pointed out, while Finnish brown hares have a dominant allele in both *DQA* and *DQB* loci, the same alleles are also abundant in mountain hares (Fig. 3). Similarly, there is evidence for gene flow from brown hare to mountain hare at the studied nuclear loci and mtDNA, although this is dwarfed by the introgression to the opposite direction (Table 1, Fig. 2). MHC loci are also notable for examples of trans-species polymorphisms^37,55^, which are known to occur even among different genera of leporids^56^. For example, TSP could explain the existence of common brown hare *DQA* and *DQB* alleles in the Finnish mountain hare population (Fig. 3, Table S3) but without samples from highly isolated populations e.g., in the arctic islands, the distinction from recent introgression cannot be easily resolved. Similarly, our phylogenetic analysis was inconclusive as the *DQA* and *DQB* alleles do not cluster as species-specific (or one species dominated) clades, unlike *UCP1*. The timing of the nodes on the tree also gave rather young approximations for the ages of the clades when compared to the speciation estimate. This is likely because the applicability of fast evolving sequences as molecular clocks saturates at deeper divergences^57^. However, the allopatric Austrian brown hares had four private *DQA* alleles and seven *DQB* allelles, while sharing only four and three alleles respectively with the Finnish hares (Fig. 3). We take this as evidence that the alleles shared among the Finnish and Austrian populations could represent TSPs or a very ancient introgression event, whereas the Finnish hares have universal allele sharing via bidirectional gene flow, as is also seen for the other investigated loci. The fact that there were no *DQA* or *DQB* private alleles in the allopatric Finnish mountain hares (Fig. 3), suggests that the Finnish mountain hares form a uniform, genetically connected population^45^ and that any beneficial alleles spread quickly in it^58^. Similarly, balancing selection involving novel allele advantage has been suggested to have promoted the introgression of MHC alleles also in other vertebrates^59–62^. As a follow-up, it would be interesting to correlate the MHC genotypes with susceptibility to specific pathogens or parasites.

In Finland, the distribution of brown hares is rather precisely limited by 150 days of snow cover, a boundary that has steadily retreated northward during the last three decades due to climate change^6^. Although capable of replacing mountain hares through direct competition^4,63^, brown hares are dependent on anthropogenic habitats in Finland and they are thought to be unable to penetrate the boreal forest ecosystems. The situation may be more complex regionally however, as brown hares can exploit road networks in their expansion and there are indications that the species may adapt to forested habitats in Nordic countries^64^. Our data give some insight into how adaptation may be occurring at the genomic level and underlines the advantage conveyed by the ability to co-opt genic variants from the resident species to open up otherwise inaccessible habitats. Climate change may facilitate this process by relaxing local selection pressures, such as the ones caused by the snow cover.

The recent decline in mountain hare numbers in Southern Finland is likely due to not only increased competition with the adapting brown hare, but also because of the shortening of the snow-covered season^44^, resulting in the camouflage mismatch of the white winter pelage and increasing predation mortality^19^. Time will tell whether Finnish mountain hare could reciprocally benefit from coat color alleles obtained from brown hare, as has been demonstrated for some other winter-white hare populations^12,13,16,18^ or if the northern hybrid brown hares are able to utilize the best of both worlds, replacing the mountain hare through much of its present range. Although the mountain hare has shown remarkable resilience in the past^65^, the extent of the combined effects of the current climate changes are likely to be complex and their outcome for the species is unknown.

## Methods

### Sample collection and DNA isolation

The Finnish hare samples were obtained from hunters and the Finnish Food Authority, as well as from a GPS/GSM telemetry study^66^. The 48 Austrian brown hare samples came from a previous study of MHC diversity in central Europe (Smith et al. 2011).

To estimate the allelic diversity and degree of introgression between the two species, we performed targeted sequencing of candidate loci likely to be under selection in hares in a total of 368 individuals: 22 allopatric mountain hares from northern Finland, 48 allopatric brown hares from the species core range in Austria and 298 sympatric specimens of both species from within the hybrid zone in central and southern Finland (Table S1). Because there are no allopatric populations of brown hares in Finland, the lowland eastern Austrian brown hares were chosen to monitor brown-hare specific allelic variation that would be free from any contemporary mountain hare genetic influence. As these hares otherwise represent a rather distant population of brown hares compared to the Finnish ones, the Austrian hares have not been included in the general population genetic analyses (genetic diversity, population structure). We also had previously obtained^9^ genotype data for 6833 SNP loci for 36 specimens of the Finnish hares (Table S1), which was used here to detect selection signatures (see below).

All specimens have been initially identified using morphological features. The species differ considerably in their habitat preferences and identification during most of the hunting season and are easily distinguished due to the white winter pelage of mountain hares. DNA from ear muscle biopsies was isolated using Chelex® 100 (Bio-Rad) method^67^, following manufacturers’ recommendations. The maps of sample locations were drawn in R v4.0.2^68^ using ggplot2^69^ and the rnaturalearth (CC BY-NC 4.0) packages.

### mtDNA genotyping

Mitochondrial DNA was genotyped for all samples (Table 1, Fig. 1, Table S1) using a previously published PCR–RFLP method^43^. Briefly, the PCR product of the mitochondrial *Cytb* locus was digested with *Dde*I, which cuts mountain hare sequence twice and brown hare sequence four times. The distinct sized restriction fragments are then unequivocally identifiable on agarose gel.

### Next generation sequencing of candidate loci

The five candidate loci analyzed in this study are: a fragment of exon 2 including the antigen binding motif of the major histocompatibility complex (MHC) class II loci *DQA* (218 bp), and *DQB* (210 bp), which have been previously reported to show both spatial as well as temporal positive selection in hares^39,48^; a 372 bp fragment of the Toll like receptor 2 corresponding to the genes for Toll-interleukin-1 receptor domain protein (*TLR2*), involved in innate immunity; a 358 bp fragment of the uncoupling protein 1 (*UCP1*); and finally a 135 bp fragment of the neutral locus, succinate dehydrogenase complex subunit A (*SDHa*). As an additional control for neutrality, we had previous genotype data for 6833 neutral SNPs from 36 specimens^9^ (Table S1). Briefly, library preparation was performed by firstly amplifying each sample using the primer pairs from Table S2. A second round of PCRs was performed to attach unique DNA barcodes to all samples and achieve compatibility with Illumina flow cell chemistry. PCR products were then purified, and after estimation of amplicon concentrations, all samples were normalized, pooled and sent to the Microsynth (AG) for sequencing on an Illumina Miseq as 250 bp paired-end. Initial amplicon data processing was achieved as outlined in Biedrzycka et al.^70^ and Sebastian et al.^71^ using the different amplicon sequencing analysis tools available at: http://evobiolab.biol.amu.edu.pl/amplisat/. Allele calls for the low polymorphism loci (*TLR2* and *SDHa*) were validated via Sanger sequencing in both directions, using the same primers as for their amplification. Genotype data for each sample is provided in Table S1. Allele frequencies and the GenBank access numbers for the sequences are given in Table S3.

### Phylogenetic analysis of candidate loci

To identify putative species-specific alleles, phylogenetic analysis was performed for *DQA*, *DQB* and *UCP1*. *TLR2* and *SDHa* were excluded as they had only three alleles each with clear-cut separation between the species (Table S3). After sequence alignment with Clustal X^72^, the analyses were performed using MrBayes^73^ with GTR + G model of evolution. The maximum-clade-credibility trees were generated using TreeAnnotator in BEAST v. 2.3.1^74,75^ and illustrated using FigTree v1.4.3^76^. Sequence clades where the majority of alleles was observed in one species only, were considered species specific.

### Genetic analyses

These analyses were conducted only on the Finnish material, representing reproductively connected hare populations. First pass data analysis, including the obtaining of standard diversity indices^36^ and testing of Hardy–Weinberg equilibria, were performed using the computational methods provided in ARLEQUIN version 3.5.2^77^. Genetic diversity (*GD*) is the measure of the number of alleles and their representation in the gene pool of the sampled population. Hardy–Weinberg equilibrium was tested using a Markov chain exact test with Forecasted chain length of 10,000 and 1000 dememorization steps.

Detection of loci under selection was performed using two different outlier detection approaches incorporating genotypes from the candidate loci combined with data from 6833 SNP loci available for a subset of 36 samples genotyped in a parallel study^9^. The first method was the *F*-statistics test based on genetic structure^40^, also provided in ARLEQUIN version 3.5.2^77^, with a hierarchical island model applying 50,000 simulations, 100 demes per group for 10 groups. The method was chosen as it allows the population sample to be assigned to different a priori groups (mountain hare, hybrid), allowing for different migrations rates between demes within groups and between groups. The latter is important because of the highly asymmetric nature of the gene flow between the two hare species. The simulation generates a joint distribution of *F*_*st*_ versus heterozygosity (Table S4). Loci that fall out of the 99% confidence intervals of the distribution are considered as being under selection. A further test of selection was conducted via the Bayesian approach of Foll and Gaggiotti (2008). The advantage of this approach is that it is amenable to small sample sizes, with a risk of low power but no particular risk of bias due to underrepresentation of sampled demes.

### Addressing trans-species polymorphisms in hares

Trans-species polymorphism (TSP), the persistence of allelic lineages across species events, should be distinguishable from introgression if the shared alleles also occur in pure parental lines of both species. This is not trivial, as it is difficult to identify isolated populations of Finnish mountain hares or brown hares to select as “pure” parental lines. Nevertheless, we conservatively identified 22 individuals that could be considered pure mountain hares: most northerly samples with mountain hare mtDNA and no brown hare characteristic alleles at *UCP1*, *TLR2* or *SDHa* loci. For brown hares, we used samples from two previous studies^39,78^ on lowland Austrian brown hares, whose populations have much longer evolutionary history of isolation from mountain hares. The samples are marked in the Table S1. Under a TSP hypothesis, we would expect little population differentiation based on population assignment due to a large number of allelic variants surviving the *Lepus timidus*/*europaeus* species diversification event, circa 5 MYA (1.2–10.1 MYA)^79–83^. We performed an Analysis of Molecular Variance (AMOVA) (Table 2) to test the level of variance within each population using GenAlEX version 6.5^84^ with 999 random permutations. BEAST v.2.5.1^85^ was used to calculate an ultrametric phylogenetic tree for each of the MHC loci and *UCP1*, implementing a HKY substitution model, a strict clock model assuming a mutation rate of 10% for DQA and 11% for DQB, and a Yule tree prior. The MCMC chain was run for 10 million steps with tree sampling every 1000 steps, and a 10% burnin fraction was used when calculating the final MCC tree with common ancestor node heights in TreeAnnotator v.1.10.4.

### Ethics declaration

No animal was killed for research purposes only and the hunting followed the regional hunting seasons and legislation (Metsästyslaki [Hunting law] 1993/615/5§). Hunted specimens did not require an ethical assessment prior to being conducted. Apart from hunted animals, some DNA samples (location Joensuu, Botania and Linnunlahti, Table S1), were obtained when animals were tagged for a separate telemetry study. Sampling caused minimal harm to the animals and was conducted following the local guidelines and under animal experimentation permission ESAVI/111/04.10.07/2014 from the Animal Experiment Board in Finland.

## Supplementary Information

Supplementary Information 1.

Supplementary Information 2.

## Data Availability

The authors affirm that all other data necessary for confirming the conclusions of the article are present within the article, figures, and tables. All data generated or analyzed during this study are included in this published article and its supplementary information files.
