# Supplementary material for: Hybridization with mountain hares increases the functional allelic repertoire in brown hares
Source: Sci Rep. 2021 Aug 4;11:15771. doi: 10.1038/s41598-021-95357-0 (PMC8338973; doi:10.1038/s41598-021-95357-0)
Supplement: Supplementary file 2 — Supplementary Information 2. [file 41598_2021_95357_MOESM2_ESM.docx]

Hybridization with mountain hares increases the functional allelic repertoire in brown hares

**SUPPLEMENTARY INFORMATION**

# Supplementary tables

**Table S1.** Sample collection details and genotyping data (Table_S1.xlsx)

**Table S2.** Primer sequences, annealing temperatures and amplicon lengths for candidate loci.

| Locus | Primer 5’-3’ | Ta (°C) | Length (bp) |
| --- | --- | --- | --- |
| DQA^1^ | F - CTTTCACTCATCAGCTGACC | 58 | 218 |
|  | R - ACAGCAGCAGTAGAGTTGGA |  |  |
| DQB^2^ | F - CCGTCCGCAGAGGATTTCGT | 58 | 210 |
|  | R - GCCTGGTAGTTGTGTCTGCA |  |  |
| TLR2^3^ | F - ATGCGTTCGTGTCCTACAGC | 58 | 372 |
|  | R - CTCAAGTTCCCCCAGAACCC |  |  |
| UCP1^4^ | F - ACCGAGTTTAGGAAGCAAGA | 52 | 358 |
|  | R - TATTTCGTTTCTCACAAGGG |  |  |
| SDHa^5^ | F - ACAAGGATCACGTCTACCTGC | 58 | 135 |
|  | R - CCCTTGTAGTTGGTAGGAATGC |  |  |

Primer sources: ^1^Gouy de Bellocq *et al.* [[1](#_ENREF_1)]; ^2^Smith *et al*. [[2](#_ENREF_2)]; ^3^ Awadi *et al.* [[3](#_ENREF_3)]; ^4^Designed for this study from an alignment with rabbit *UCP1* (acc. # NM001171077); ^5^Designed for this study from an alignment with brown hare *SDHa* (acc. # DQ402979).

**Table S3.** Allele frequencies and the GenBank accession numbers for the sequenced loci. #novel allelic variant discovered in this study. Frequencies for the Finnish hare population only.

| **Allele** | ***timidus*** | ***europaeus*** | **GenBank #** |
| --- | --- | --- | --- |
| Leeu-DQA*04  Leeu-DQA*07  Leeu-DQA*09  Leeu-DQA*12  Leeu-DQA*010 | 0.12 | Austria  Austria  Austria  Austria  0.40 | EU686541.1  EU876910.1  EU876912.1  EU876915.1  EU876913.1 |
| Leeu-DQA*001 | 0.18 | 0.19 | EU686543.1 |
| Leeu-DQA*034 | 0.19 | 0.14 | EU876937.1 |
| Leti-DQA*004 | 0.09 | 0.03 | EU686549.1 |
| Leti-DQA*005 | 0.09 | 0.01 | EU686548.1 |
| Leeu-DQA*011 | 0.03 | 0.00 | EU876914.1 |
| #Leti-DQA*006 | 0.07 | 0.00 | MK719589 |
| Leeu-DQA*006 | 0.01 | 0.16 | EU876909.1 |
| #Leti-DQA*007 | 0.07 | 0.02 | MK719590 |
| #Leti-DQA*008 | 0.14 | 0.02 | MK719591 |
| Leti-DQA*001 | 0.01 | 0.01 | EU686550.1 |
| Leeu-DQA*008 | 0.00 | 0.01 | EU876911.1 |
| #Leeu-DQB*002  #Leeu-DQB*003  #Leeu-DQB*005  #Leeu-DQB*006  #Leeu-DQB*008  #Leeu-DQB*009  #Leeu-DQB*010  #Leeu-DQB*001 | 0.08 | Austria  Austria  Austria  Austria  Austria  Austria  Austria  0.54 | MK719558.1  MK719559.1  MK719561.1  MK719562.1  MK719564.1  MK719565.1  MK719566.1  MK719557 |
| #Leti-DQB*001 | 0.08 | 0.16 | MK719568 |
| #Leti-DQB*002 | 0.10 | 0.01 | MK719569 |
| #Leti-DQB*003 | 0.10 | 0.03 | MK719570 |
| #Leti-DQB*004 | 0.05 | 0.02 | MK719571 |
| #Leti-DQB*005 | 0.00 | 0.07 | MK719572 |
| #Leeu-DQB*007 | 0.01 | 0.06 | MK719563 |
| #Leti-DQB*006 | 0.05 | 0.01 | MK719573 |
| #Leti-DQB*007 | 0.04 | 0.02 | MK719574 |
| #Leti-DQB*008 | 0.07 | 0.00 | MK719575 |
| #Leti-DQB*009 | 0.04 | 0.00 | MK719576 |
| #Leti-DQB*010 | 0.07 | 0.01 | MK719577 |
| #Leti-DQB*011 | 0.03 | 0.00 | MK719578 |
| #Leeu-DQB*004 | 0.03 | 0.00 | MK719560 |
| #Leti-DQB*012 | 0.09 | 0.01 | MK719579 |
| #Leti-DQB*013 | 0.03 | 0.01 | MK719580 |
| #Leti-DQB*014 | 0.00 | 0.00 | MK719581 |
| #Leti-DQB*015 | 0.01 | 0.00 | MK719582 |
| #Leti-DQB*016 | 0.06 | 0.01 | MK719583 |
| #Leti-DQB*017 | 0.00 | 0.00 | MK719584 |
| #Leti-DQB*018 | 0.00 | 0.00 | MK719585 |
| #Leti-DQB*019 | 0.01 | 0.00 | MK719586 |
| #Leti-DQB*020 | 0.00 | 0.02 | MK719587 |
| #Leti-DQB*021 | 0.05 | 0.01 | MK719588 |
| #SDH01 | 0.05 | 0.72 | MK719592 |
| #SDH02  #SDH03 | 0.95 | 0.28  Austria | MK719593  MW239648 |
| #TLR2_01 | 0.11 | 0.63 | MK719601 |
| #TLR2_02  #TLR2_03 | 0.89 | Austria  0.37 | MK719602  MW239649 |
| #UCP01 | 0.76 | 0.38 | MK719594 |
| #UCP02 | 0.00 | 0.28 | MK719595 |
| #UCP03  #UCP04  #UCP05 | 0.07 | 0.29  Austria  Austria | MK719596  MW239642  MW239643 |
| #UCP06  #UCP07  #UCP08  #UCP09 | 0.02 | 0.04  Austria  Austria  Austria | MK719597  MW239644  MW239645  MW239646 |
| #UCP10 | 0.11 | 0.01 | MK719598 |
| #UCP11  #UCP13 | 0.02 | Austria  0.00 | MK719599  MW239646 |
| #UCP14 | 0.02 | 0.01 | MK719600 |

**Table S4.** Observed (Obs.) heterozygosity (*Hz*), *F^ST^* and *F^ST^* *p*-values for the sequenced loci in Finnish hares, as obtained from F-statistic based selection analysis applying hierarchical island model, 100 simulated demes and 50,000 coalescent simulations. Median value is given for the SNPs. Loci below the 99% confidence intervals (1- FST quantile >0.99) of the distribution are considered as being under selection.

| **Locus** | **Obs. *Hz* BP** | **Obs *F^ST^*** | ***F^ST^* *p*-value** | **1- *F^ST^* quantile** |
| --- | --- | --- | --- | --- |
| *DQA* | 0.88 | 0.08 | 1.15e-031 | 1.00 |
| *DQB* | 0.93 | 0.14 | 1.22-009 | 1.00 |
| *UCP1* | 0.69 | 0.19 | 0.01 | 0.99 |
| *TLR2* | 0.61 | 0.54 | 0.37 | 0.62 |
| *SDHa* | 0.75 | 0.64 | 0.33 | 0.66 |
| SNPs | 0.94 | 0.91 | 0.31 | 0.31 |

# References

1. Gouy de Bellocq, J., et al., *Evolutionary history of an MHC gene in two leporid species: characterisation of Mhc-DQA in the European brown hare and comparison with the European rabbit.* Immunogenetics, 2009. **61**(2): p. 131-44.

2. Smith, S., et al., *Evolutionary genetics of MHC class II beta genes in the brown hare, Lepus europaeus.* Immunogenetics, 2011. **63**(11): p. 743-751.

3. Leroy, G., et al., *Inbreeding impact on litter size and survival in selected canine breeds.* Veterinary Journal, 2015. **203**(1): p. 74-78.
